# Supplementary material for: Molecular Characterization of Tc964, A Novel Antigenic Protein from Trypanosoma cruzi
Source: Int J Mol Sci. 2020 Mar 31;21(7):2432. doi: 10.3390/ijms21072432 (PMC7177413; doi:10.3390/ijms21072432)
Supplement: Supplementary file 1 [file ijms-21-02432-s001.pdf]

Supplementary files

Table S1. Tc964 gene sequences reported in different DTUs and strains from *T. cruzi*

ID Gene TrytripD	Accession number GenBank	Strain	(DTU)	Reference
TcCLB.511467.70 Curated ref. strain	XP_811413	CL_Brener_ Esmeraldo-like	VI	[27]
C4B63_54g169	PWU89982	Dm28c2018	I	[28]
BCY84_19579	PBJ69506	Dm28c2017	I	[29]
TCDM_03713	ESS67641	Dm28c2014	I	[30]
TCSYLVIO_000964	EKG07898.1	Sylvio_X10	I	[31]
TcCLB.506295.50	XP_812855	CL_Brener_Non-Esmeraldo-like	VI	[27]
Tc_MARK_3808	EKF3246.1	marinkellei_strain_B7	Tcbat	[32]

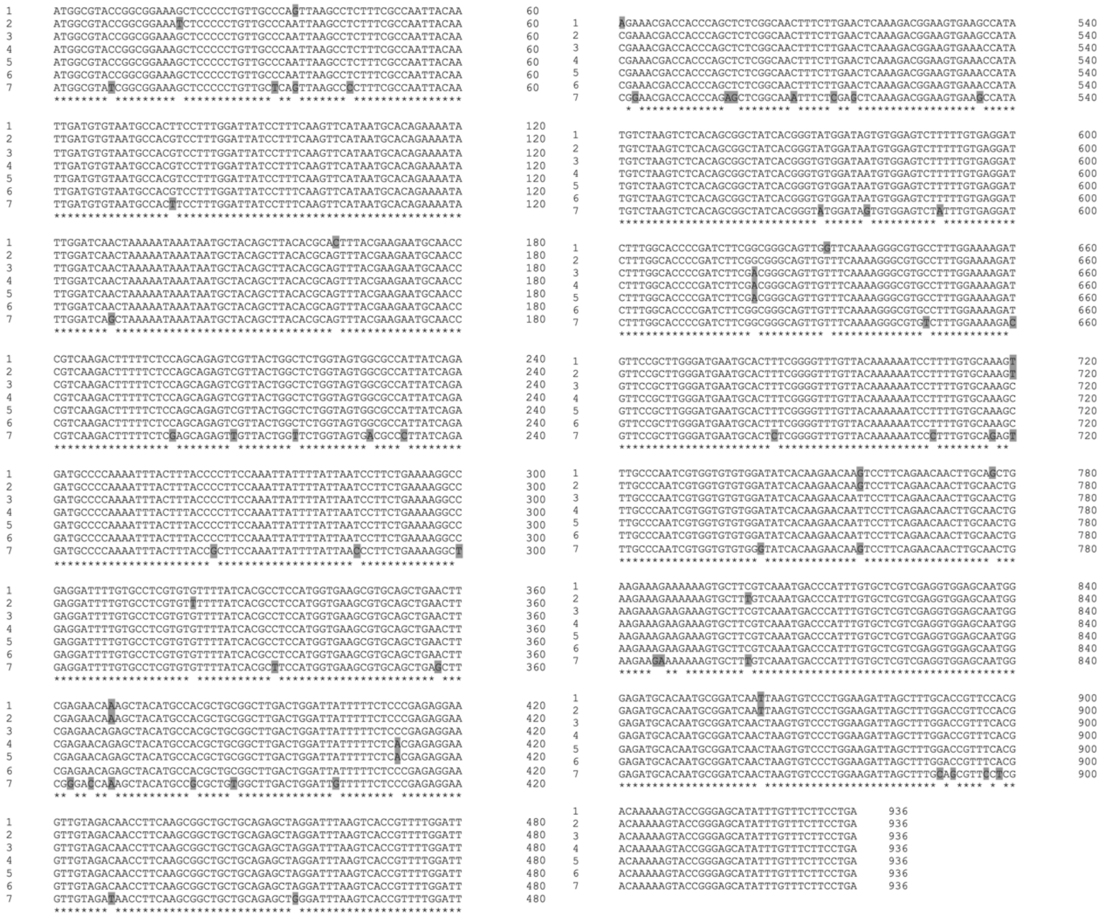

Figure S1. Multiple alignments between the *T. cruzi* sequences reported in the TrytripDB database for the *TCSYLVIO_000964* gene (*T. cruzi* Sylvio_X10), with the Omega Clustal program [44]. The sequences are identified as: **1.** *T. cruzi* CL Brener Esmeraldo Like (DTU VI) reference strain **2.** *T. cruzi* CL Brener Non-Esmeraldo Like (DTU VI) **3.***T. cruzi* Dm28c 2014 (DTU I) **4.** *T. cruzi* Dm28c 2017 (DTU I) **5.** *T. cruzi* Dm28c 2018 (DTU I) **6.** *T. cruzi* Sylvio_X10 (DTU I) **7.** *T. cruzi* marinkellei strain B7 (DTU VII). The polymorphisms detected between strains, are highlighted in gray. (*) Identical bases.

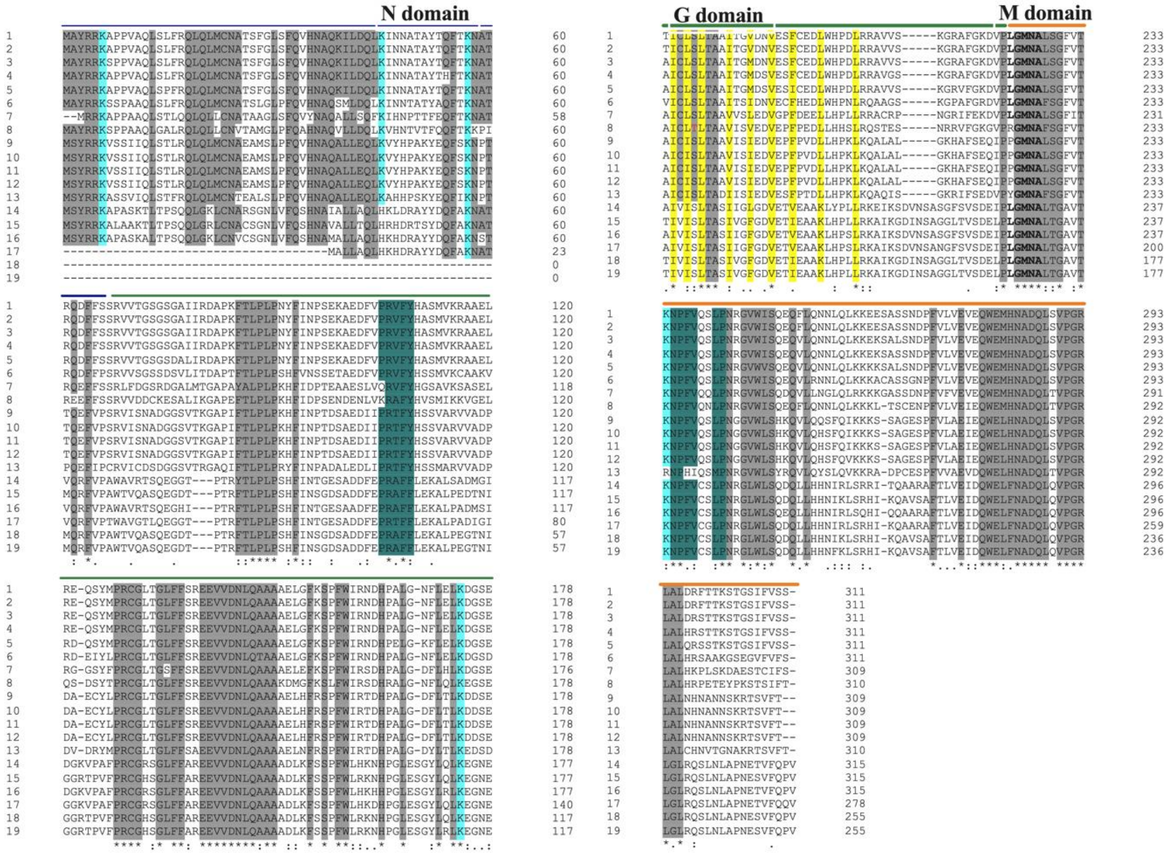

Figure S2. Multiple alignment between the kinetoplastid sequences reported in the TrytripDB database and NCBI for the Tc964 protein, with the Omega Clustal program [44]. The sequences are identified as: **1.** *T. cruzi* Dm28c 2018 (DTU I) **2.** *T. cruzi* Sylvio_X10 (DTU I) **3.** *T. cruzi* CL Brener Non-Esmeraldo Like (DTU VI) **4.** *T. cruzi* CL Brener Esmeraldo Like (DTU VI) **5.** *T. cruzi* marinkellei strain B7 (DTU VII) **6.** *T. rangeli* **7.** *T. grayi* **8.** *T. theileri* **9.** *T. brucei* TREU927 **10.** *T. brucei* gambiense **11.** *T. brucei* Lister 427 **12.** *T. evansi* **13.** *T. congolense* **14.** *L. infantum* **15.** *L. braziliensis* **16.** *L. major* Friedlin **17.** *L. mexicana* **18.** *L. panamensis* **19.** *L. guyanensis*. Identical or similar sequence blocks are highlighted in gray. (*) Identical amino acids, (:) conserved substitutions, (.) less conserved substitutions. Domains Tc964 protein are located on sequence. The (PY-NLS) motifs are highlighted in green and the (NES) is highlighted in yellow.

© 2020 by the authors. Submitted for possible open access publication under the terms and conditions of the Creative Commons Attribution (CC BY) license (http://creativecommons.org/licenses/by/4.0/).
